# Left axillary cannulation for acute type A aortic dissection

**DOI:** 10.1186/s13019-022-01928-1

**Published:** 2022-08-20

**Authors:** Yang-Xue Sun, Mao-Long Meng, Gang Li, Hong-Wei Guo

**Affiliations:** 1 Department of Surgery, Fuwai Hospital, National Center for Cardiovascular Diseases, Chinese Academy of Medical Sciences and Peking Union Medical College, 167# Beilishi Road, Beijing, 100037 China; 2 Department of Surgery, Shenzhen Hospital, Fuwai Hospital, Chinese Academy of Medical Sciences, Shenzhen, China

**Keywords:** Type A aortic dissection, Axillary artery, Femoral artery, Cardiopulmonary bypass, Cannulation

## Abstract

The most commonly used arterial cannulation sites for type A aortic dissection are right axillary artery, femoral artery and both. Direct central aortic cannulation has also been reported. In rare cases, it is extremely difficult to choose an arterial cannulation site for type A aortic dissection due to involvement of the right axillary and both femoral arteries. Herein, we present a 39-year-old male with acute type A aortic dissection with involvement of the right axillary and both femoral arteries. Left axillary cannulation was made and selective cerebral perfusion was performed through direct left common carotid artery cannulation during circulatory arrest. Surgery was performed to replace the ascending aorta and total arch combined with a frozen elephant trunk implantation. The patient recovered uneventfully. To our knowledge, this is a rare case of total aortic arch replacement with frozen elephant trunk implantation through left axillary arterial cannulation for type A aortic dissection in the literature. Left axillary cannulation is a safe and useful choice for type A aortic dissection surgery when right axillary and femoral cannulation are not safe and reliable.

## Introduction

Left axillary cannulation is very rarely used for type A aortic dissection, as right axillary, femoral artery and both are commonly and safely used [1–5]. Nevertheless, it is a choice for type A aortic dissection with involvement of the right axillary and both femoral arteries. Herein, we present a 39-year-old male of acute type A aortic dissection with involvement of the right axillary and both femoral arteries. Left axillary cannulation, ascending aorta and total arch replacement combined with a frozen elephant trunk implantation was performed.

## Case report

**Fig. 1:**
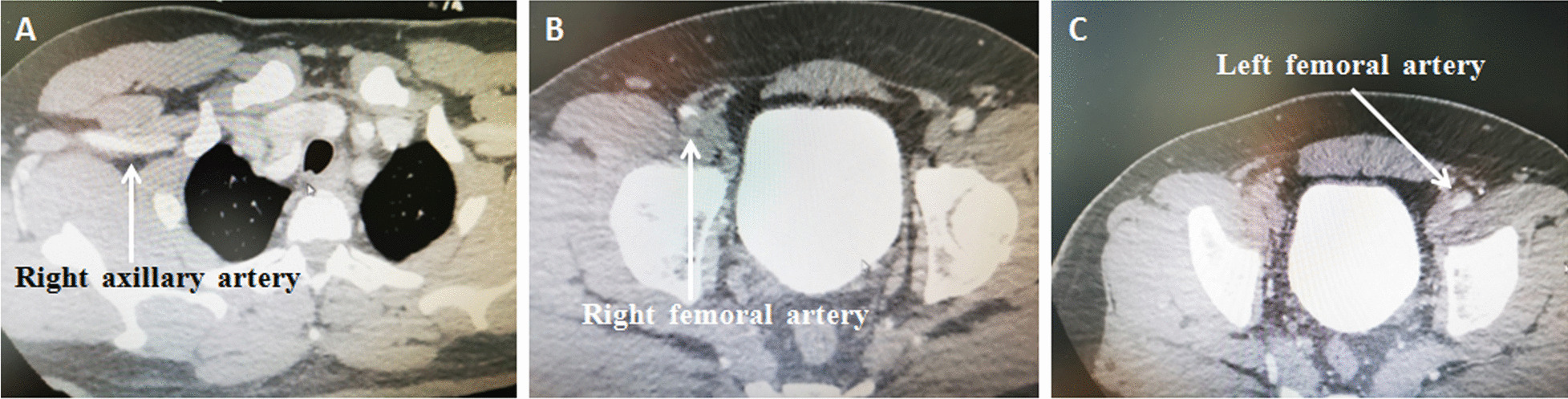
Acute type A aortic dissection with involvement of the right axillary artery (**A**), right femoral artery (**B**), and left femoral artery (**C**)

**Fig. 2:**
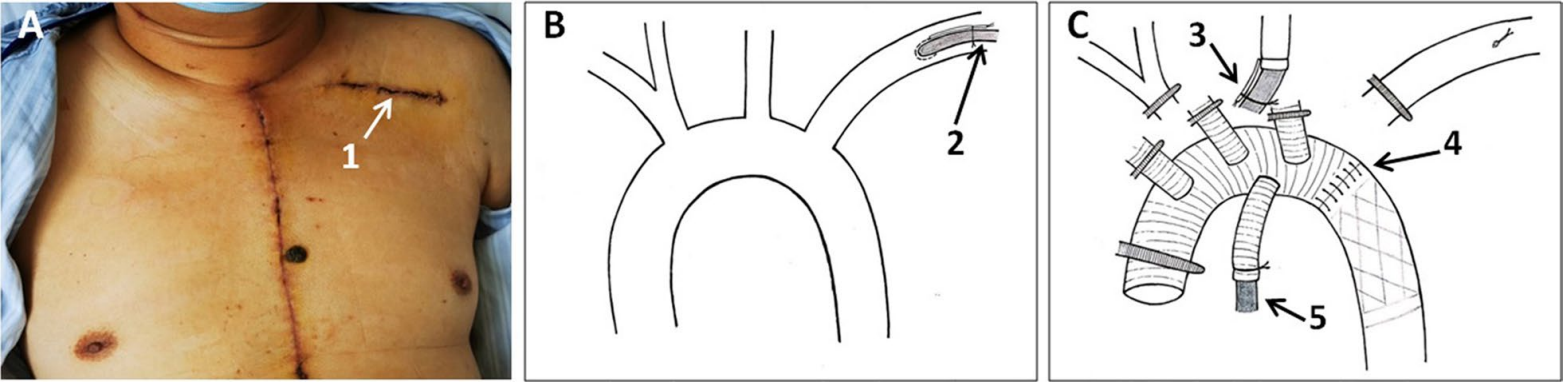
**A.1** Skin incision for left axillary cannulation; **B.2** Left axillary cannulation; **C.3** Direct left common carotid artery cannulation for selective cerebral perfusion during circulatory arrest; **C.4** The distal main of four branches prosthetic vessel was anastomosed to the elephant trunk and descending aorta; **C.5** The left axillary cannulation was transferred to the perfusion branch of the four branches prosthetic vessel. Distal perfusion was restored and circulatory arrest ended

This study has been approved by the Ethics Committee of the Shenzhen Fuwai Hospital (SP20211101(01)). A 39-year-old male was admitted to our hospital with sudden chest and back pain for 9 h. Computed tomographic (CT) scan showed type A aortic dissection involving the right axillary artery and both femoral arteries (Fig. 1). Surgery was performed through a median sternotomy. Cardiopulmonary bypass (CPB) was established with left axillary (Fig. 2A, B), right atrial cannulation, and left heart venting from the right superior pulmonary vein. After the patient was cooled down to 28 °C, the incision was extended onto the ascending aorta and transected the branchiocephalic arteries, selective cerebral perfusion was performed with antegrade and unilateral perfusion from direct left common carotid artery cannulation (Fig. 2C). The aortic arch was opened and a frozen elephant trunk (CRONUS 26 mm × 100 mm) was installed into the descending aorta. The distal main of four branches prosthetic vessel (MAQUET HEMASHIELD PLATINUM 28/10/8/8 × 10 mm) was anastomosed to the elephant trunk and descending aorta (Fig. 2C). The left axillary cannulation was transferred to the perfusion branch of the four branches of the prosthetic vessel; distal perfusion was restored and circulatory arrest ended (Fig. 2C).

The branchiocephalic arteries are separate sutured to the branches of the prosthetic vessel, total aortic arch and reconstruct the branches of the aortic arch in the following order: the branchiocephalic artery, the left common carotid artery, the left subclavian artery. After the branchiocephalic artery was sutured to the polyester graft, removing air and particulate matter by flow from perfusion branch, antegrade selective right brain perfusion was initiated. When the left common carotid artery, bilateral antegrade brain perfusion was restored.

The cardiopulmonary bypass time was 264 min, cross-clamp time was 149 min, and circulatory arrest time was 20 min. The patient recovered uneventfully. No neurological deficit or hoarseness were observed. The duration of mechanical ventilation support was 89.1 h. The duration of stay in the intensive care unit was 206 h. The postoperative hospital stay was 26 days.

## Discussion

The right axillary artery, femoral artery, and both are the most commonly used cannulation sites for type A aortic dissection in current practice [1–4]. Direct central aortic cannulation has also been suggested by some groups [5]. Fast, safe and reliable arterial cannulation for CPB establishment is essential for acute type A aortic dissection surgery. This case study demonstrates in those rare cases when the right axillary artery and both femoral arteries are involved, and aortic dissection and cannulation in these sites are not safe and reliable, left axillary cannulation is an option.

In this case, a 39-year-old male was diagnosed as acute type A aortic dissection involving right axillary artery and both femoral arteries. Left axillary cannulation was made for CPB establishment. Ascending aorta, total arch and frozen elephant trunk implantations were performed. The patient recovered uneventfully and was discharged on day 26 post-operation.

## Conclusion

Left axillary cannulation for type A aortic dissection surgery is safe and reliable. Left axillary cannulation is an option for type A aortic dissection surgery when right axillary and both femoral arteries are not safe and reliable for arterial cannulation.

## Data Availability

The datasets used during the current study are available from the corresponding author on reasonable request.

## Abbreviations

CT: Computed tomographic
CPB: Cardiopulmonary bypass
